# Case report: First report of haploidentical allogeneic hematopoietic stem cell transplantation from donors with mild alpha-thalassemia for acute leukemia

**DOI:** 10.3389/fonc.2022.986144

**Published:** 2022-12-08

**Authors:** Wenshuai Zheng, Yamei Wu, Lixun Guan, Longcan Cheng, Yalei Hu, Min Tan, Yuhui Yang, Hongmei Ning

**Affiliations:** ^1^ Department of Hematology, Hainan Hospital of Chinese PLA General Hospital, Sanya, China; ^2^ Department of Hematology, Seventh Medical Center of Chinese PLA General Hospital, Beijing, China; ^3^ Senior Department of Hematology, Fifth Medical Center of Chinese PLA General Hospital, Beijing, China

**Keywords:** haploidentical donors, thalassemia, acute leukemia, successful, haploidentical allogeneic hematopoietic stem cell transplantation

## Abstract

For acute leukemia (AL) with adverse prognostic factors, allogeneic hematopoietic stem cell transplantation (allo-HSCT) is the standard care option after the first complete remission. Meanwhile, as the success of haploidentical HSCT (haplo-HSCT), haploidentical donors (HIDs) become a reliable choice. However, there have been no reports on haplo-HSCT from HIDs with mild alpha(α)-thalassemia for AL yet. In the present report, we first describe two cases of successful haplo-HSCT from HIDs with mild α-thalassemia for AL.

## Introduction

Acute leukemia (AL) is a heterogeneous disease characterized by impaired differentiation and increased proliferation of myeloid progenitor cells (1). Allogeneic hematopoietic stem cell transplantation (allo-HSCT) is the standard care option for AL with adverse prognostic factors (2, 3). For the donor selection of allo-HSCT, human leukocyte antigen (HLA)-matched sibling donors (MSDs) are generally preferred, with haploidentical donors (HIDs) and matched unrelated donors (MUDs) as alternatives (4). In China, due to the shortage of MSDs and MUDs, haploidentical HSCT (haplo-HSCT) has achieved great success (5). Nevertheless, in areas where thalassemia is endemic, it is inevitable that part of the HIDs have thalassemia. Currently, the safety and efficacy of haplo-HSCT from HIDs with mild alpha(α)-thalassemia for AL is unknown. We first report two cases of AL patients received haplo-HSCT from HIDs with mild α-thalassemia.

## Case presentation

Two patients were diagnosed as AL and received haplo-HSCT from HIDs with mild α-thalassemia. The patients charts were screened for patient history and actual clinical data and laboratory findings. Both patients were female, aged 18 and 56 years with HIDs. The detailed patients’ and donors’ characteristics are shown in **Table 1**. Patient 1 was diagnosed as mixed phenotype acute leukemia (MPAL) with two blast populations of distinct lineages (B lymphoid/myeloid) and had multiple gene mutations with poor prognosis. Patient 2 was diagnosed as acute myeloid leukemia (AML) classified as high risk group. We considered allo-HSCT for them and performed HLA typing and blood tests of their family members to find a suitable donor. For patient 1, we fund two HIDs and no MSDs and MUDs were identified. Her elder son is a HID with positive hepatitis B virus (HBV) infection (HBV DNA = 1.84 × 10 (6)/ml) and younger son is a HID with mild anemia. The detection of thalassemia revealed that her younger son is a patient with mild α-thalassemia. Since her elder son had positive HBV infection and younger son had hematological stability with hemoglobin (Hb) levels maintained at approximately 11-12 g/dL, we recommended her younger son as HID of haplo-HSCT. For patient 2, HLA typing showed that her father, mother and little brother are HIDs and no MSDs and MUDs were identified. Her father and little brother also had mild anemia with Hb levels maintained at approximately 10-12 g/dL. The detection of thalassemia revealed that both of her father and little brother are patients with α-thalassemia. Because her family refused to use her mother as donor, we choosed the younger donor as her HID. The course of therapy, conditioning regimen and graft-versus-host disease (GVHD) prophylaxis can be seen in **Table 1**. Peripheral blood stem cells (PBSCs) were harvested from HIDs after four days of granulocyte colony stimulating factor (G-CSF). For patient 1, the total nucleated cell, CD34+, and CD3+ cell counts were 8.9 × 10(8)/kg, 7.1 × 10(6)/kg, and 2.9 × 10(8)/kg, respectively. White blood cell (WBC) was engrafted on +14 day and platelet was engrafted on +12 day (The day of WBC engraftment was defined as the first of three consecutive days on which the granulocyte count exceeded 0.5 × 10 (8)/L. The day of platelet engraftment was defined as the first of seven consecutive days on which the platelet count exceeded 20 × 10(8)/L without platelet infusion). For patient 2, the total nucleated cell, CD34+, and CD3+ cell counts were 7.97 × 10(8)/kg, 9.12 × 10(6)/kg, and 2.5 × 10(8)/kg, respectively. Both WBC and platelet were engrafted on +9 day. In both patients, regimen-associated toxicities, such as anorexia, enteritis and infection, were mild and there were II degree cutaneous acute GVHD which was controlled by first-line treatment with glucocorticoids. The 1, 2, 3, 6 month follow-up of patient 1 and 1, 2, 3, 6, 9, 12, 18, 24, 36 month follow-up of patient 2 showed that bone marrow examination were normocellular marrow, and chimerism analysis by variable-number tandem repeat were full donor chimerism. No chronic GVHD were observed. The detection of thalassemia showed that both patients converted to donors’ thalassemia type with mild microcytic hypochromic anemia in which Hb levels maintained at approximately 10-12 g/dL without transfusion.

**Table 1:** The clinic characteristics of patients and donors.

Patients	Age	Diagnosis	Karyotype	Mutation	Treatment and remission	Conditioning regimen	Immunosuppression	Donors	Age	Sex	Thalassemia type
Patient 1	56	MPAL	46, XX[20]	*IDH2*, *NRAS*, *WT1*, *EZH2*	DA+VP, NR DA+AZA, CR MRD(-) DA+AZA, CR MRD(-) DA+AZA, CR MRD(-)	Fludarabine (30 mg/m^2^ -6 to -3d) Carmustine (300 mg/m^2^ -6d) Busulfan (0.8 mg/kg/6h -5 to -3d)	Tacrolimus (0.015 mg/kg/12h from +5d with drug level monitoring) Cyclophosphamide (50mg/kg +3 to +4 d) MMF (1g/12h +5 to +28d)	Donor 1	30	Man	- -^SEA/^αα
Patient 2	18	AML	45, X, -X, t (8: 21) (q22: q22), arr (4) × 3[20]	*C-kit*	DA, PR IAC, CR MRD(+) DA, CR MRD(-)	Busulfan (0.8 mg/kg/6h -10 to -8d) Cytarabine (4 g/m^2^ -6 to -5d); Cyclophosphamide (50 mg/kg -4 to -3d) Anti-thymoglobulin (1.5 mg/kg -5d, 2.5 mg/kg -4 to -3d, 3.5 mg/kg -2d)	CSA (2 mg/kg/12h from -10d with drug level monitoring) MTX (10 mg/m^2^ +1, +3, +6, and +11d) MMF (1g/12h -10d to +28d)	Donor 2	11	Man	- -^SEA/^αα

AML, acute myeloid leukemia; AZA, azacitidine; CR, complete remission; CSA, cyclosporine A; DA, daunorubicin + cytarabine; GVHD, graft-versus-host disease; IAC, Idarubicin+cytarabine+cladribine; MRD, minimal residual disease; MMF, mycophenolate mofetil; MPAL, mixed phenotype acute leukemia; MTX, methotrexate; NR, non-remission; PR, partial remission; VP, vincristine+prednison.

## Discussion

The treatment of AL involves initial induction therapy and post-remission therapy. The goal of post-remission therapy is to prevent relapse of the disease. The two commonly strategies of post-remission are additional post-remission cytotoxic chemotherapies or allo-HSCT. The choice of therapy is determined by the unique risks and benefits provided by each treatment.

In our cases, patient 1 was diagnosed as MAPL. Clinically, the outcomes for MPAL are worse than both acute lymphoblastic leukemia (ALL) and AML (7). In addition, patient 1 didn’t get CR after first course chemotherapy. Patient 2 has karyotype of monomer, which was classified as high risk group. So we recommended allo-HSCT for them to decrease the choice of relapse. But both patients were lack of suitable MSDs and MUDs. Under the circumstances, haplo-HSCT is an alternative option. A systematic review showed that the relapse, survival, and acute GVHD were not significantly different between MSD-HSCT and haplo-HSCT using post transplantation cyclophosphamide (PT-Cy) (9). Wang et al. reported that the haplo-HSCT and MSD-HSCT groups exhibited comparable 3-year non-relapse mortality, disease free survival and overall survival in intermediate/high-risk AML (6).

In our cases, because of some reasons as above, we choosed the younger son of patient 1 and the little brother of patient 2 as donor, however, both donors had mild α-thalassemia. In areas where thalassemia is endemic, this is an inevitable situation sometimes. According to the suitability criteria for adult related donors, hematopoietic stem cell collections from individuals with red blood cell abnormalities such as spherocytosis and elliptocytosis are generally not recommended; however, subjects with mild α-thalassemia, or β-thalassemia are suitable hematopoietic stem cell donors (8). Nevertheless, to our best knowledge, no case of AL patients undergoing haplo-HSCT from HIDs with mild α-thalassemia have been reported yet. Despite having α-thalassemia phenotype, the two donors had sufficiently stable hematology. After careful consideration, we opted haplo-HSCT for them.

α-thalassaemia is inherited as an autosomal recessive disorder characterised by a microcytic hypochromic anaemia, and a clinical phenotype varying from almost asymptomatic to a lethal haemolytic anaemia (10). It is probably the most common monogenic gene disorder in the world and is especially frequent in Mediterranean countries, South-East Asia, Africa, the Middle East and in the Indian subcontinent (10). Mild α-thalassemia is usually asymptomatic but sometimes involves mild anemia which does not require treatment (10). However, considering the pathophysiology of α-thalassemia, our main concern was the risk of poor engraftment of erythrocytes after HSCT and the efficiency of the mobilization of PBSCs in patients with thalassemia. Mi YJ et al. reported a rare case in which allo-HSCT for a 7-year-old severe aplastic anemia patient from a MSD with mild β-thalassemia was performed successful. In this case, the engraftment of erythrocytes was evident, good performance status has been observed throughout the 5 years after HSCT and the PBSCs mobilized by G-CSF were sufficient (11). Li K et al. also reported that for the efficiency of mobilization of PBCSs in patients with thalassemia, there were no significant differences in the CD34+ cell subsets and lymphocyte subsets after G-CSF administration when compared with those in healthy donors (12).

In our cases, PBSCs were harvested from donors’ peripheral blood after four days of G-CSF administration to the day of transplantation. The total nucleated cell and CD34+ cell counts were sufficient and the donors had no complication. Both patients’ WBC and platelet were engrafted successfully and no further blood transfusions have been required to this day.

## Conclusion

Our findings suggest that HIDs with mild α-thalassemia could be suitable donors of allo-HSCT for AL and the mobilization and collection of PBSCs in patients with mild α-thalassemia are feasible.

## Data availability statement

The datasets presented in this study can be found in online repositories. The names of the repository/repositories and accession number(s) can be found in the article/supplementary material.

## Ethics statement

Written informed consent was obtained from the individual(s) for the publication of any potentially identifiable images or data included in this article.

## Author contributions

WZ and HN conceived and designed the study, interpreted the results, wrote the paper and gave critical comments. LC, YH, MT, YY collected the data. YW and LG wrote the paper and gave critical comments. All authors contributed to the article and approved the submitted version.
